# The human microbiome: an emerging tool in forensics

**DOI:** 10.1111/1751-7915.12699

**Published:** 2017-02-27

**Authors:** Jarrad T. Hampton‐Marcell, Jose V. Lopez, Jack A. Gilbert

**Affiliations:** ^1^ Biosciences DivisionArgonne National LaboratoryLemontIL60443USA; ^2^ Department of Biological SciencesUniversity of Illinois at ChicagoChicagoIL60607USA; ^3^ The Microbiome CenterUniversity of ChicagoChicagoIL60637USA; ^4^ Department of Biological SciencesNova Southeastern UniversityFort LauderdaleFL33314USA; ^5^ Department of SurgeryUniversity of ChicagoChicagoIL60637USA

Advances in sequencing technology have enabled DNA profiling to become a staple in criminal forensics. Short tandem repeats (STRs) embedded in an individuals’ genetic code enable authorities to take advantage of biological variability to accurately identify and discriminate among people. According to the National DNA Index System (NDIS), CODIS, a DNA database containing more than 12 million profiles, has assisted in more than 340,000 criminal investigations in the USA (CODIS ‐ NDIS Statistics, n.d.). However, this still represents a small percentage of total crimes committed. Indeed, many criminal cases still go unsolved despite advances in DNA profiling; for example, in 2015, only 20% of residential burglaries (>1.5 million) were resolved by authorities, according to the 2015 FBI Uniform Crime Reporting statistics (Clearances, n.d.). This can be explained in part by resource allocation, as burglaries are not prioritized for investigation compared to other higher profile crimes (Paré *et al*., 2007; Coupe, 2016), which can lead to significantly reduced response times, resulting in crime scene evidence contamination or destruction, further impeding investigative efficiency. Therefore, there is a need to improve the lines of evidence that can be acquired to link perpetrators to the crime scene.

Improving trace evidence options for criminal investigations is a major focus for forensic research specialists globally. One possible option that has recently emerged encompasses the symbiotic microorganisms that reside in and on our bodies. The NIH‐funded ‘Human Microbiome Project’ (HMP) has significantly improved the scientific and public recognition of the vital importance of symbiont ecology to host health and development (Consortium, 2012; Methé *et al.,* 2012; Grice, 2015). There are approximately as many bacterial cells in our body as human cells (Sender *et al*., 2016) and the compliment of bacterial taxa, especially at the subspecies level, appears to be unique to each person (Zhu *et al*., 2015) offering a compelling opportunity to develop a new identifiable marker unique to the individual. The microbiome is even unique in identical twins (Goodrich *et al*., 2014), theoretically offering an opportunity to increase identity resolution over that possible with human genome evidence. However, the microbiome changes over time in an individual (Oh *et al*., 2016), so how can it be used to identify a person? While the relative proportions of the bacteria do indeed change, the composition of the community appears to be relatively stable (Caporaso *et al*., 2011; David *et al*., 2014), although this stability and continued identifiability are areas of active research. Interestingly, the fluctuations in the structure and composition of the microbiome may contain useful information that could also be used for forensic purposes. Host lifestyle, including diet, occupation, travel, and pharmaceutical use, can influence the composition and structure of microbiome. This suggests that profiling the microbial community in and on our body could also help to reveal details about an individual's lifestyle (Gonzalez *et al*., 2016; Kuntz and Gilbert, 2017), which could represent new trace evidence.

Profiling the microbiome may be useful in identifying a person or their lifestyle characteristics, but for burglary, the microbiome of the perpetrator would need to be detected at the crime scene, in their absence, while retaining the identifiable characteristics. In support of this, we know that humans shed ~30 million bacterial cells into their vicinity every hour (Qian *et al*., 2012) and researchers have already demonstrated the forensic potential of the microbiota left behind by people on physical surfaces. For example, the bacterial community found on your finger tips (microbial fingerprint) could be traced on a keyboard, so that which keyboard, and even which keys, a person used could be identified based on the bacterial residue (Fierer *et al*., 2010). Furthermore, mobile phones carry the personal microbial signatures of the owner (Meadow *et al*., 2014; Lax *et al*., 2015). Importantly, these are just preliminary studies, and the results and conclusions cannot be used to justify the application of microbial sequencing to forensic studies. However, the statistical basis for the accurate matching of a person to their microbiota, and evidence that a residual microbial fingerprint could be used to discriminate individuals, does suggest that in the future, it may be possible to use these profiles for forensic investigations. Yet, however intimate the association, because microbial compositions can shift with environmental factors and over time, they cannot be definitively equated with the host genome profile. Therefore, a substantial amount of research is still required to prove that residual microbial fingerprints can be used as effective trace evidence. This is currently being undertaken as part of a National Institute of Justice programme of awards that focus on research into the use of the microbiome and metabolome for forensic investigation.

While microbiome profiling could potentially serve as a complement to human DNA profiling, it is not clear whether the microbiome can scale across institutions using forensic‐based evidence due to the data resource requirements and the associated costs for maintaining these databases. DNA profiling still needs to be matched to a subject, and additional trace evidence can help to narrow the search for a perpetrator, which creates a need for profiling techniques that are not limited to CODIS. For serial burglaries, modus operandi behaviour has shed some light by modelling various crime scenes (Markson *et al*., 2010). While helpful, it is limited to frequency and geography, which could be influenced by a number of different factors. Researchers have shown that cosmetics, antibiotics usage, dietary profiles, and even health states have been associated with corresponding changes in the skin microbiota (Rosenthal *et al*., 2011). Citizen science‐based research initiatives like the Human Microbiome Project and others (Huttenhower *et al*., 2012; Methé *et al*., 2012) are creating vast data resources that can help to predict host–microbiome relationships that could improve our ability to predict specific lifestyle characteristics based on a residual microbial signature.

While human microbial fingerprinting should never replace traditional DNA profiling techniques, there is the possibility that in the future, it could help augment existing trace evidence options for forensic researchers. This will require substantial investment in standardization and implementation of microbiome profiling techniques, as well as the development of detection technologies that could automate or rapidly advance microbiome profiling. Such innovation necessitates new enterprise and job creation, which can help to advance the translation of this burgeoning area of science. The potential to integrate bioinformatists and microbial ecologists as paid position in forensic laboratories is not as far‐fetched as it might seem at first glance. The authors have visited the lead forensic laboratory in China, and have seen first‐hand that microbiome science is already being used to advance trace evidence for criminal activity. One such example included matching the microbial profile of residual soil on a shovel owned by a suspect to the grave site of a murder. By collecting soil samples from hundreds of sites in the area, it was possible to predict with an extremely high probability that the soil of the shovel came from the grave site based entirely on the microbiome profile. Even though there is much work to be done, having a microbial database that complements CODIS could prove an effective method to lowering crime rates and clearing cases.
